# Crystal structure and Temperature-Dependent Luminescence Characteristics of KMg_4_(PO_4_)_3_:Eu^2+^ phosphor for White Light-emitting diodes

**DOI:** 10.1038/srep09673

**Published:** 2015-04-09

**Authors:** Jian Chen, Yangai Liu, Lefu Mei, Haikun Liu, Minghao Fang, Zhaohui Huang

**Affiliations:** 1 Beijing Key Laboratory of Materials Utilization of Nonmetallic Minerals and Solid Wastes, National Laboratory of Mineral Materials, School of Materials Science and Technology, China University of Geosciences, Beijing, 100083

## Abstract

The KMg_4_(PO_4_)_3_:Eu^2+^ phosphor was prepared by the conventional high temperature solid-state reaction. The crystal structure, luminescence and reflectance spectra, thermal stability, quantum efficiency and the application for N-UV LED were studied respectively. The phase formation and crystal structure of KMg_4_(PO_4_)_3_:Eu^2+^ were confirmed from the powder X-ray diffraction and the Rietveld refinement. The concentration quenching of Eu^2+^ in the KMg_4_(PO_4_)_3_ host was determined to be 1mol% and the quenching mechanism was certified to be the dipole–dipole interaction. The energy transfer critical distance of as-prepared phosphor was calculated to be about 35.84Å. Furthermore, the phosphor exhibited good thermal stability and the corresponding activation energy Δ*E* was reckoned to be 0.24eV. Upon excitation at 365nm, the internal quantum efficiency of the optimized KMg_4_(PO_4_)_3_:Eu^2+^ was estimated to be 50.44%. The white N-UV LEDs was fabricated via KMg_4_(PO_4_)_3_:Eu^2+^, green-emitting (Ba,Sr)_2_SiO_4_:Eu^2+^, and red-emitting CaAlSiN_3_:Eu^2+^ phosphors with a near-UV chip. The excellent color rendering index (Ra = 96) at a correlated color temperature (5227.08K) with CIE coordinates of *x* = 0.34, *y* = 0.35 of the WLED device indicates that KMg_4_(PO_4_)_3_:Eu^2+^ is a promising blue-emitting phosphor for white N-UV light emitting diodes (LEDs).

Since the first light emitting diode (LED) light source was invented by Nick Holonyak of General Electric, it has drawn more and more attention to apply in solid-state lighting and create an enormous revolution on the lighting industry. Recently, a great attention has been focused on white LEDs as solid-state lighting and as components of display devices because of their low energy consumption, high efficiency, long operational lifetime (>100 000h), environmental friendliness and high material stability^1^,^2^,^3^,^4^. There are several ways to assemble the white LEDs. The most prevalent strategy is produced by pumping the blue InGaN chip with yellow-emitting Y_3_Al_5_O_12_:Ce^3+^ (YAG) phosphor. Nevertheless, high correlated color temperature (CCT) and low color rendering (CRI) index (Ra < 80) restrict it to provide sunlight-like illumination due to the deficiency in red emission. In order to generate excellent CRI values and appropriate CCT white light for display or general illumination light sources, the method of pumping blue, green, and red-emitting phosphors with near ultraviolet (N-UV) LEDs has been investigated^5^,^6^,^7^. Thereinto, the BaMgAl_10_O_17_:Eu^2+^ (BAM), which is the most commonly used commercial blue phosphor for N-UV LEDs as high efficiency, suffer from poor thermal stability^4^,^8^,^9^. Accordingly, the development of excellent structure and thermal stability of blue phosphor host for N-UV LEDs is highly desirable.

Eu^2+^ ion is the most frequently used blue-emitting activator in phosphor, which shows broad N-UV excitation and visible emission in a specific host owing to the 4*f*–5*d* transitions. According to the impact of the strength of the crystal field and covalent, Eu^2+^ can emit light from the ultraviolet to the infrared with broadband emitting fluorescence in different matrixes, and the corresponding fluorescence lifetimes locate commonly in the range of 0.2–2.0μs^10^,^11^. On basis of these characteristics, multifarious Eu^2+^ -activated phosphors have been widely studied in LED lighting, 3D displays and scintillators in detection devices.

The phosphate KMg_4_(PO_4_)_3_ compound was first obtained from flux during crystallizing K_2_MgWO_2_(PO_4_)_2_ by Tomaszewski and co-workers^12^. Xiaofeng Lan et al. reported the luminescence properties of Eu^2+^-activated KMg_4_(PO_4_)_3_ by combustion-assisted synthesis method in 2012^13^. However, to our best knowledge, the temperature-dependent luminescence characteristics as well as the application of KMg_4_(PO_4_)_3_:Eu^2+^ pumped for *n*-UV LEDs have not been investigated. In this paper, the KMg_4_(PO_4_)_3_:Eu^2+^ phosphor was firstly prepared by the conventional high temperature solid-state reaction method. The crystal structure, reflectance spectra, thermal stability, quantum efficiency and applications in white NUV LED are studied respectively. White LEDs was fabricated by combing an N-UV LED chip (λ_max_ = 385nm) with the KMg_4_(PO_4_)_3_:Eu^2+^, along with green and red phosphors, and its optical properties have also been investigated. The results demonstrate that the blue-emitting KMg_4_(PO_4_)_3_:Eu^2+^ is a promising blue-emitting phosphor for white N-UV LEDs.

## Results

### XRD Refinement and Crystal Structure

**Figure 1:**
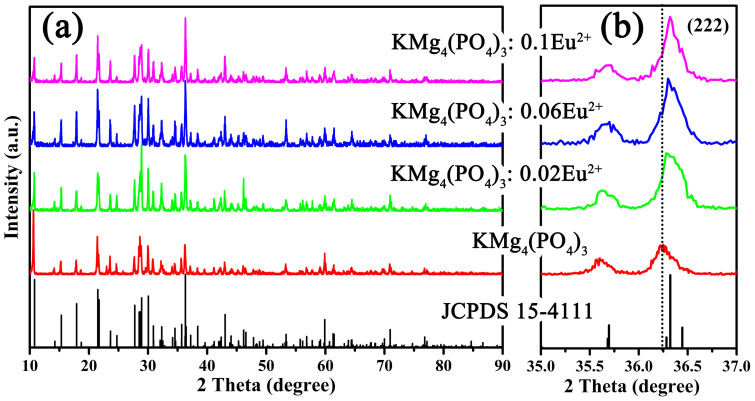
(a) XRD patterns of the as-prepared samples KMg_4_(PO_4_)_3_:*x*Eu^2+^ (*x* = 0, 0.02, 0.06, and 0.1) and the standard pattern (JCPDS 15-4111). (b) The detailed XRD patterns ranging from 10° to 12° for the same samples.

**Figure 2:**
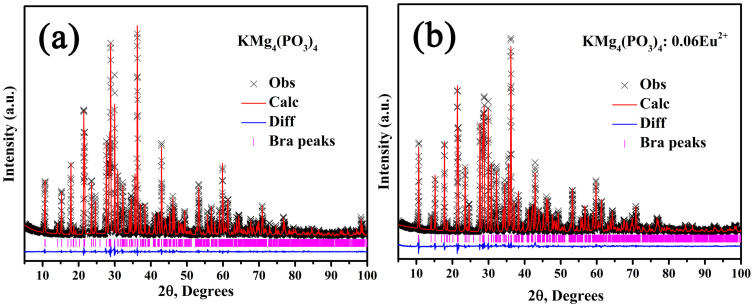
Powder XRD pattern (×) of (a) KMg_4_(PO_4_)_3_ host and (b) KMg_4_(PO_4_)_3_:0.06Eu^2+^ samples with their corresponding Rietveld refinement (solid line) and residuals (bottom).

Figure 1 depicts the XRD patterns of the series of as-synthesized KMg_4_(PO_4_)_3_:*x*Eu^2+^ (*x* = 0, 0.02, 0.06, and 0.1), and the standard pattern (JCPDS 15-4111) of KMg_4_(PO_4_)_3_ is shown as a reference. It can be found from the Figure 1 that all XRD patterns agree well with the standard pattern and no other phase is observed, which demonstrates that the single phase of KMg_4_(PO_4_)_3_:*x*Eu^2+^ was obtained and the doping of Eu^2+^ ions did not cause any notable impurities or any structural variation. Besides, the main diffraction peaks shift slightly to the higher angle side with increasing Eu^2+^ concentrate, as shown in Figure 1(b). This observation means that the lattice was distorted by substitution the ions which are comparatively big radius in KMg_4_(PO_4_)_3_ host lattice with Eu^2+^ ions^14^. Thus, it is reasonable to assume that Eu^2+^ (r = 1.25Å for coordinate number (CN) = 8 and r = 1.17Å for CN = 6) ions substituted the position of K^+^ sites (r = 1.51Å for CN = 8) because both the Mg^2+^ (r = 0.72Å for CN = 6 and r = 0.66Å when CN = 5) and P^5+^ (r = 0.17Å for CN = 4) sites are smaller than the Eu^2+^ ions^15^,^16^. For further understanding the phase purity and the occupancy of Eu^2+^ ions on K^+^ sites in KMg_4_(PO_4_)_3_:Eu^2+^, the Rietveld refinement of KMg_4_(PO_4_)_3_ and KMg_4_(PO_4_)_3_:0.06Eu^2+^ phosphors were analyzed via the GSAS program as shown in Figure 2. The KMg_4_(PO_4_)_3_ was served as an initial structural model. The results of Rietveld refinement further demonstrate that neither the host nor the doped 0.06mol Eu^2+^ ions generated any impurity or secondary phases in KMg_4_(PO_4_)_3_. The KMg_4_(PO_4_)_3_:*x*Eu^2+^ belongs to an orthorhombic structure with the space group Pnnm(58). For the crystal of KMg_4_(PO_4_)_3_ host, the lattice parameters were fitted to be *a* = 16.3707(7)Å, *b* = 9.5627(4)Å, *c* = 6.1667(5)Å, cell volume (V) = 965.361(23)Å^3^ and the weighted profile R-factor (R_wp_), the expected R factor (R_p_) are 8.86% and 6.86%, respectively. As doped with Eu^2+^, the lattice parameters of KMg_4_(PO_4_)_3_:0.06Eu^2+^ became *a* = 16.3563(4)Å, *b* = 9.5570(2)Å, *c* = 6.1663(1)Å and V = 963.904(67)Å^3^. The refinement data converged to R_wp_ = 10.54% and R_p_ = 7.97%, as summarized in Table 1. The volumetric constriction with increasing Eu^2+^ doping concentration also indicates that the Eu^2+^ occupied the K^+^ ions sites.

**Figure 3:**
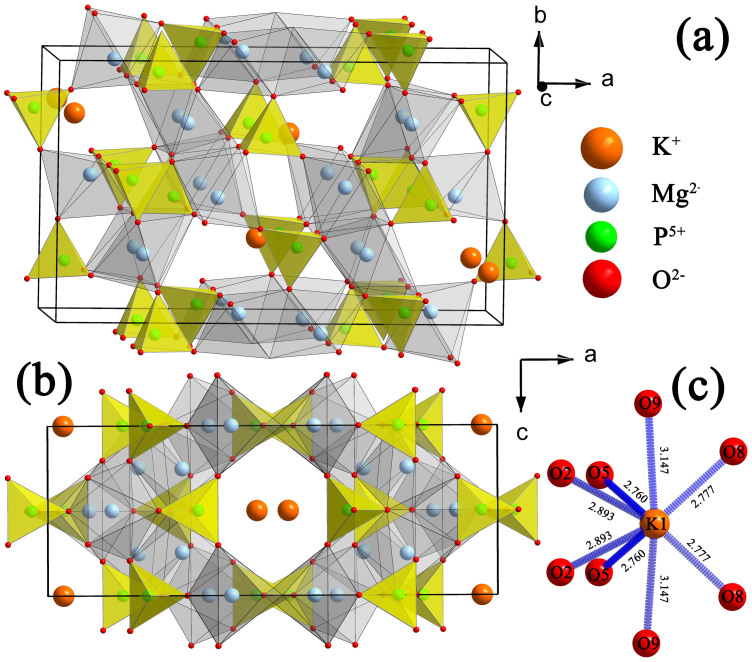
Crystal structures of KMg_4_(PO_4_)_3_. The view of the KMg_4_(PO_4_)_3_ (a) along the [001] Direction, (b) perpendicular to the [010] direction, and the (c) coordination geometry of anions around the K^+^ ions.

Figure 3 illustrates the crystal structure of KMg_4_(PO_4_)_3_ and the coordination environment of the K^+^ ions. The compound of KMg_4_(PO_4_)_3_ is consist of PO_4_ tetrahedra, MgO_6_ octahedra and MgO_5_ polyhedra which are linked by P–O–Mg bridges. The K^+^ ions are located in the tunnels along b-axis and surrounded by the three-dimensional framework forming via interconnected polyhedra. As presented in Figure 3c, the K^+^ ions in KMg_4_(PO_4_)_3_ are eight-fold coordinated by oxygen ions with 4g position and m site symmetry.

### Reflectance and Photoluminescence properties of the KMg_4_(PO_4_)_3_:*x*Eu phosphor at RT

**Figure 4:**
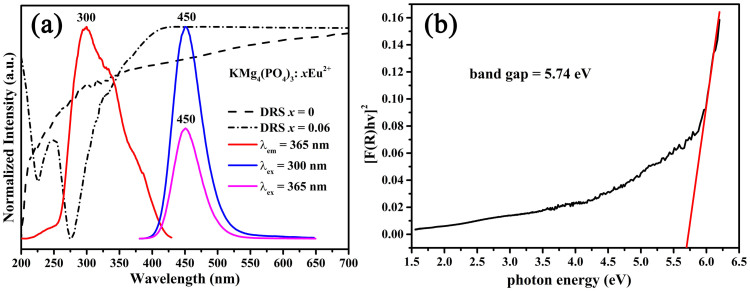
(a) Excitation and emission spectra of KMg_4_(PO_4_)_3_:0.06Eu^2+^ (λ_em_ = 450nm for excitation and λ_ex_ = 300 and 365nm for emission); Diffuse reflection spectra of KMg_4_(PO_4_):*x*Eu^2+^ (*x* = 0 and 0.06); (b) Absorption spectra of KMg_4_(PO_4_)_3_:0.06Eu^2+^ matrix calculated by the Kubelka−Munk equation. All spectra were taken at RT.

The reflectance spectra of KMg_4_(PO_4_)_3_ host and KMg_4_(PO_4_)_3_:0.05Eu^2+^ are presented in Figure 4a. The KMg_4_(PO_4_)_3_ host shows an energy absorption band ranging from 200 to 300nm, and a high reflection ranging from 300 to 700nm. The band gap of the virgin KMg_4_(PO_4_)_3_ is calculated by using the following formula:^17^
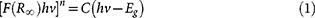
in which *hν* means the energy per photon, C is a proportional constant and E_g_ represents the value of the band gap, n = 1/2 stands for an indirect allowed transition, 2 means a direct allowed transition, 3/2 represents a direct forbidden transition, or 3 indicates an indirect forbidden transion, R_∞_ = R_sample_/R_standard_. The F(R∞) means the Kubelka−Munk function which can be formulated to the following equation:
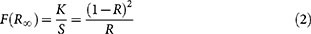
where K, S, and R represent the absorption, scattering, and reflectance parameter, respectively. As illustrated in Figure 4b, the band gap energy of KMg_4_(PO_4_)_3_ host is estimated to be about 5.74eV from the extrapolation of the line for [F(R∞)hν]^2^ = 0. As Eu^2+^ ions were introduced into the host, strong broad absorption appeared in the 250–400nm N-UV range, which is matched well with the excitation spectrum.

**Figure 5:**
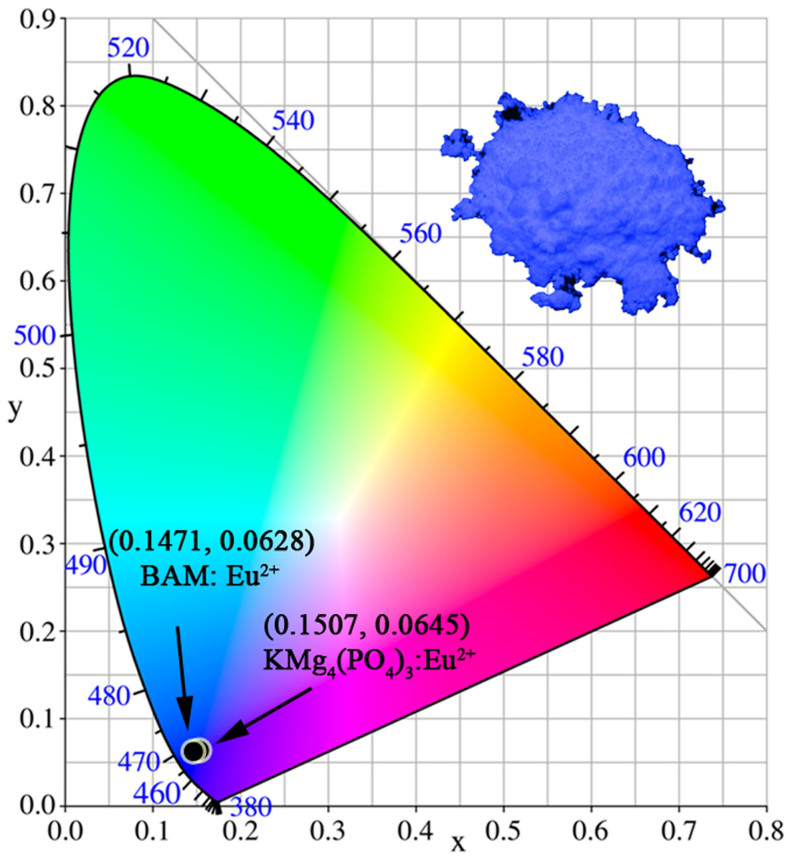
CIE coordinates of the KMg_4_(PO_4_)_3_:0.01Eu^2+^ phosphor and the commercial BAM:Eu^2+^. The inset shows a digital photograph of the blue-emitting KMg_4_(PO_4_)_3_:0.01Eu^2+^ sample.

The photoluminescence emission (PL, λ_ex_ = 300 and 365nm) and excitation (PLE, λ_em_ = 450nm) spectra of KMg_4_(PO_4_)_3_:0.06Eu^2+^ are also depicted in Figure 4a. The PLE spectrum of KMg_4_(PO_4_)_3_:0.06Eu^2+^ presents a broad hump ranging at 250–400nm, which originates from the 4*f*^7^–4*f*^6^5*d* transition of Eu^2+^ ions. It indicates that the broad excitation spectrum of KMg_4_(PO_4_)_3_:Eu^2+^ matches well with the emission of the commercial N-UV chip (365–420nm). The PL spectra of the KMg_4_(PO_4_)_3_:0.06Eu^2+^ phosphor present a 52.2nm full width at half-maximum (FWHM) broad blue emission band extending from 400 to 525nm peaking at 450nm, which is assigned to the 4*f*^6^5*d*–4*f*^7^ transition of the Eu^2+^ ions. Moreover, The PL of KMg_4_(PO_4_)_3_:0.06Eu^2+^ detected under 365nm is similar to that under 300nm in addition to the difference of the relative intensity, which verifies that the Eu^2+^ ions occupy the same lattice site (K^+^ sites) in KMg_4_(PO_4_)_3_ host^18^. Above result is in accord with the conclusion from Rietveld refinement. The CIE chromaticity coordinates of KMg_4_(PO_4_)_3_:0.01Eu^2+^ and commercial BAM phosphors under 365nm UV excitation are illustrated in Figure 5. The color coordinates of KMg_4_(PO_4_)_3_:0.01Eu^2+^ and BAM are calculated to be (*x* = 0.1507, *y* = 0.0645) and (*x* = 0.1471, *y* = 0.0628), respectively. The inset shows the digital photograph of KMg_4_(PO_4_)_3_:0.01Eu^2+^ phosphor under a 365nm UV lamp, which indicates KMg_4_(PO_4_)_3_:Eu^2+^ phosphor can be used as a blue-emitting phosphor for *w*-LEDs application.

**Figure 6:**
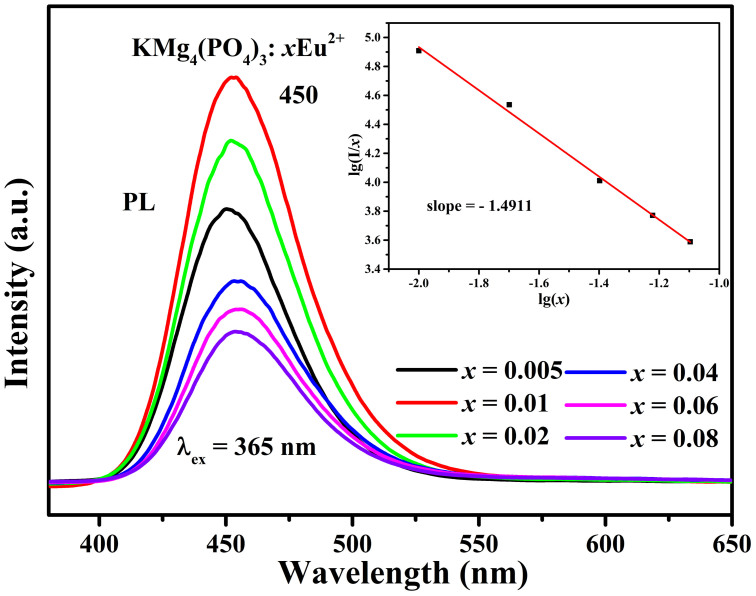
PL spectra for KMg_4_(PO_4_)_3_:*x*Eu^2+^ (*x* = 0.005–0.08) phosphors. The inset shows the fitting line of lg(*I/x*) versus lg(*x*) in KMg_4_(PO_4_)_3_:*x*Eu^2+^ (*x* = 0.005–0.08) phosphors beyond the quenching concentration.

As Figure 4a shows, the PL and PLE spectra of KMg_4_(PO_4_)_3_:0.06Eu^2+^ overlap partially, which demonstrates the existence of energy transfer between Eu^2+^-Eu^2+^ 19. As we know, two main mechanisms can be explanatory for the resonant energy-transfer: exchange interaction or electric multipolar interaction^20^. In order to further investigate the process of energy transfer between activators or between sensitizer and activator, the Eu^2+^ concentration-dependent PL spectra of KMg_4_(PO_4_)_3_:*x*Eu^2+^ (*x* = 0.005, 0.01, 0.02, 0.04, 0.06, 0.08) phosphors under 365nm light excitation are shown in Figure 6. The optimal doping concentration of Eu^2+^ for the PL intensity in KMg_4_(PO_4_)_3_:*x*Eu^2+^ is 1% mol. When the doping content of Eu^2+^ exceeded 0.01mol%, the PL intensity began to decrease because of the concentration quenching effect which is due to the energy consumed via energy transfer from one activator to another^21^. Thus, the critical distance (Rc) for energy transfer among Eu^2+^ is necessary to obtain for further understanding the concentration quenching interaction mechanism. The value of the critical distance (Rc) can be reckoned via the following equation:^22^
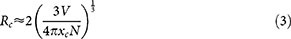
where *V* means the unit cell volume, *x_c_* represents the concentration of activator ion where the quenching occurs and *N* is the number of the K^+^ ion in per unit cell. For the KMg_4_(PO_4_)_3_ host, *x_c_* = 0.01, *N* = 4, and *V* = 965.361Å^3^, hence, the value of R_c_ is calculated to about 35.84Å. Owing to the typical critical distance of the exchange interaction is about 5Å and the exchange interaction only fits the energy transfer of forbidden transitions^5^,^23^. Therefore, the electric multipolar interactions are dominant in the energy transfer process. The interaction type can be estimated via the following equation:^24^
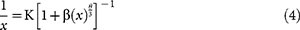
in which *x* is the concentration of activation, which is not less than the critical concentration, *I/x* is the emission intensity (*I*) per activator concentration (*x*); K and β are constants under the same excitation condition of host lattice; and *θ* is a function of electric multipolar character. *θ* = 6, 8, 10 for dipole–dipole (*d*–*d*), dipole–quadrupole (*d*–*q*), quadrupole–quadrupole (*q*–*q*) interactions, respectively. In order to estimate the θ value, the dependence of lg(*I/x*) on lg(*x*) is illustrated in the inset of Figure 6. A relatively linear relation can be observed and the slope of the straight line is fitting to −1.4911 which equals to −*θ*/3. Hence, the value of *θ* is 4.4733, which is close to 6, demonstrating that the interaction type in KMg_4_(PO_4_)_3_:Eu^2+^ is dipole-dipole interactions.

**Figure 7:**
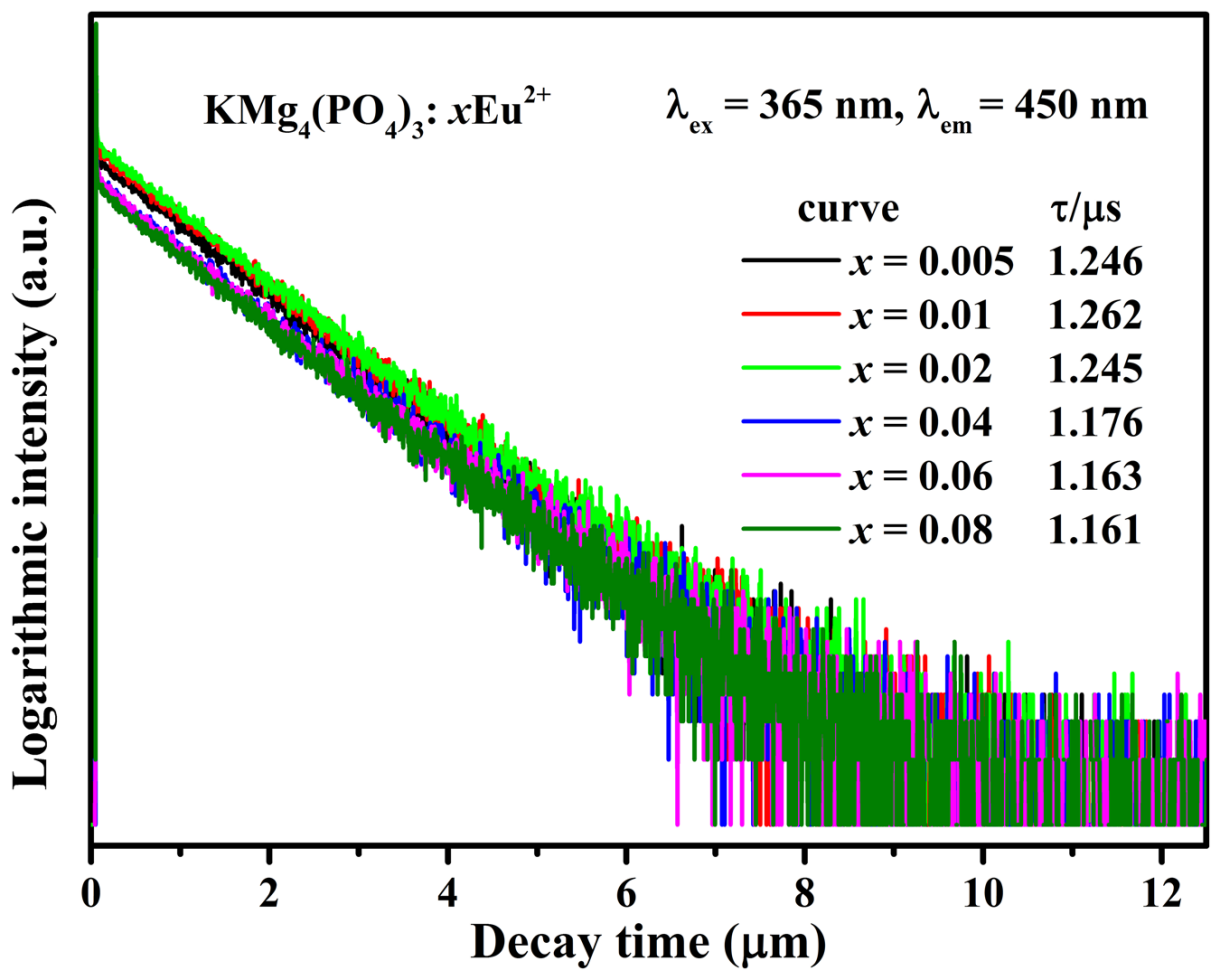
Decay lifetime tests of KMg_4_(PO_4_)_3_:*x*Eu^2+^ (x = 0.05–0.08) detected at 450nm for Eu^2+^ emission.

To further explore the energy transfer process, the room temperature luminescence decay curves of Eu^2+^ ions in KMg_4_(PO_4_)_3_:*x*Eu^2+^ (*x* = 0.005, 0.01, 0.02, 0.04, 0.06, 0.08; λ_ex_ = 365nm, λ_em_ = 450nm) were measured, as shown in Figure 7. The decay curves can be fitted with an approximate single-exponential decay model as:
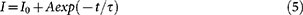
where *I_0_* represents the initial emission intensity when *t* is 0 and *τ* means the lifetime. The average lifetimes of Eu^2+^ ions of KMg_4_(PO_4_)_3_:*x*Eu^2+^ (*x* = 0.005, 0.01, 0.02, 0.04, 0.06, 0.08) were estimated to be 1.246, 1.262, 1.245, 1.176, 1.163 and 1.161μs, respectively. All measured decay times are reasonable for the 5*d*–4*f* allowed transition of Eu^2+^ in solids (~1μs). Besides, the decay time increases to maximum, when the concentration of Eu^2+^ increases to 0.01, and then the decay time reduces acutely, indicating an efficient energy transfer between Eu^2+^ ions and causing concentration quenching^25^,^26^.

### Influence of Temperature on emission intensity and FWHM

**Figure 8:**
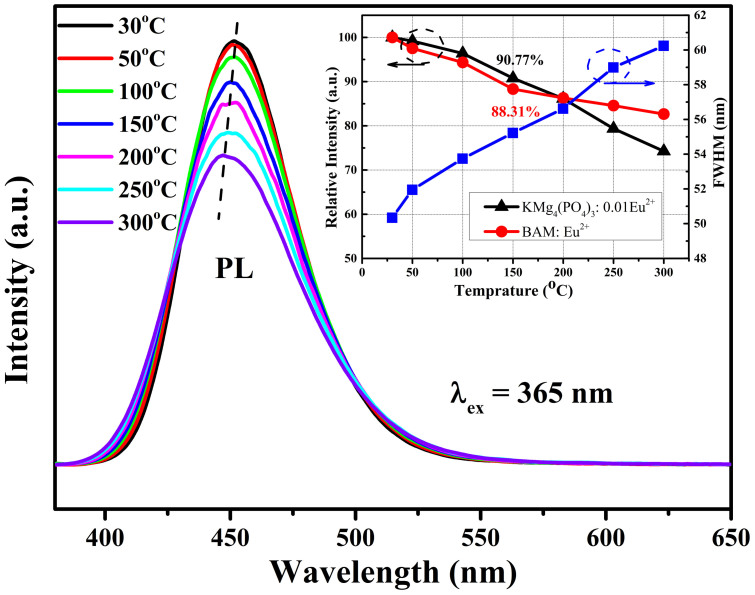
Temperature-dependent PL spectra of KMg_4_(PO_4_)_3_:0.01Eu^2+^. The inset shows a comparison of thermal quenching of KMg_4_(PO_4_)_3_:0.01Eu^2+^ with that of BAM:Eu^2+^ and dependence of the FWHM of KMg_4_(PO_4_)_3_:0.01Eu^2+^ emission on temperature.

**Figure 9:**
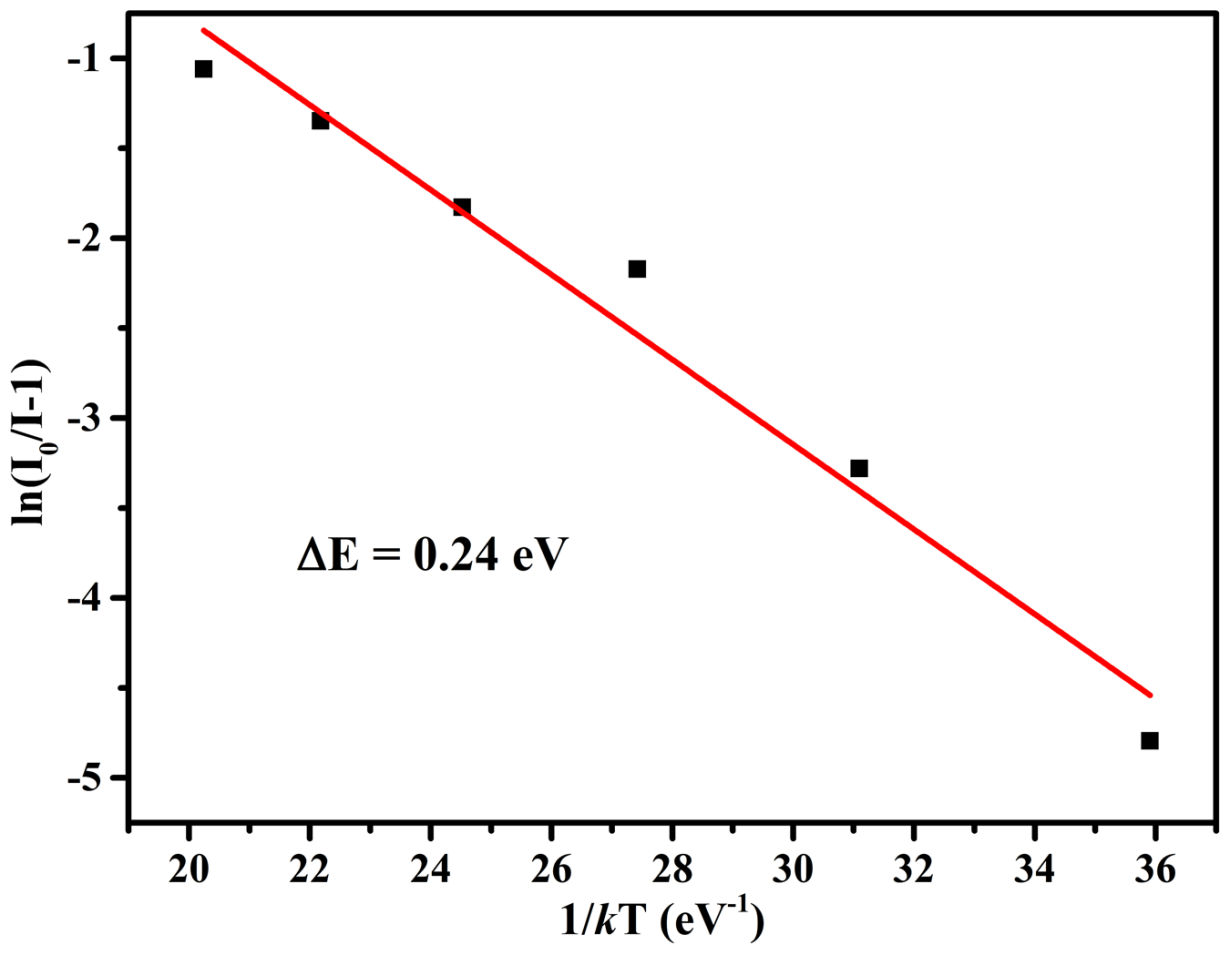
The plot of ln (*I0/I*) vs. *1/T* of the KMg_4_(PO_4_)_3_:0.01Eu^2+^ phosphors.

The thermal quenching of luminescence is one of important technological parameters to be considered for phosphor materials applied in high power LEDs, because it significantly affects the light output and service life. The temperature-dependent PL spectra of the KMg_4_(PO_4_)_3_:0.01Eu^2+^ exited by 365nm N-UV light is depicted in Figure 8. With increasing temperature (30°C–300°C), the emission intensity decreases from 100% to 74.26% of that at 30°C, and the FWHM of the emission band increases from 50.34 to 60.42nm. The inset of Figure 8 illustrates the comparison of the thermal luminescence quenching of KMg_4_(PO_4_)_3_:0.01Eu^2+^ with that of commercial BAM:Eu^2+^ and the FWHM of KMg_4_(PO_4_)_3_:0.01Eu^2+^ emission as a function of the temperature. As shown in Figure 8, it can be found only 9% decay at 150°C for KMg_4_(PO_4_)_3_:0.01Eu^2+^, which indicates that the thermal stability of KMg_4_(PO_4_)_3_:Eu^2+^ is superior to that of commercial BAM:Eu^2+^ below 200°C and this phosphor could be used as a promising phosphor for high-power LED application. To better understand the thermal quenching process, the configurational coordinate diagram can be used to respond to this phenomenon. As the temperature increases, the interaction of electron-phonon is intensive. Along with the enhancing of phonon interaction, more electrons can be thermally activated to the crossover between the 4*f*^6^5*d* excited state and 4*f*^7^ ground state, whereupon release the energy by generating lattice vibration^22^. The excellent thermal stability of KMg_4_(PO_4_)_3_:Eu^2+^ may relate to the indurative structure which is combined with PO_4_ tetrahedra, MgO_6_ octahedra and MgO_5_ polyhedra via P-O-Mg bridges to form the three-dimensional framework. In this indurative structure, the energy of the electrons in the excited state is difficult to release via lattice vibration, which means smaller Stokes shift in a configurational coordinate diagram model and higher activation energy (Δ*E*)^27^. The activation energy can be estimated by using the Arrhenius equation:^28^
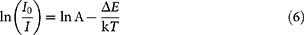
in which *I_0_* and *I* are the luminescence intensity of KMg_4_(PO_4_)_3_:Eu^2+^ at room temperature and a given temperature, respectively; A is a constant; k is the Boltzmann constant (8.617 × 10^−5^eV K^−1^). From above equation, the Δ*E* is calculated to be about 0.24eV (Figure 9).

The temperature-dependent of emission FWHM is related to the configuration coordinate model and the Boltzmann distribution, and can be expressed by:^18^,^29^
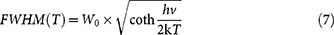

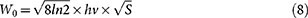
where *W_0_* is the FWHM at 0°C, *hv* represents the vibrational phonon energy, S means the Huang−Rhys parameter, and k is the Boltzmann constant. With the temperature increase, the excited electrons spread to higher vibration levels and the radiative transitions from these different levels cause the emission band broadening.

### Quantum efficiency and Electroluminescence properties of White-Light LED Lamp

**Figure 10:**
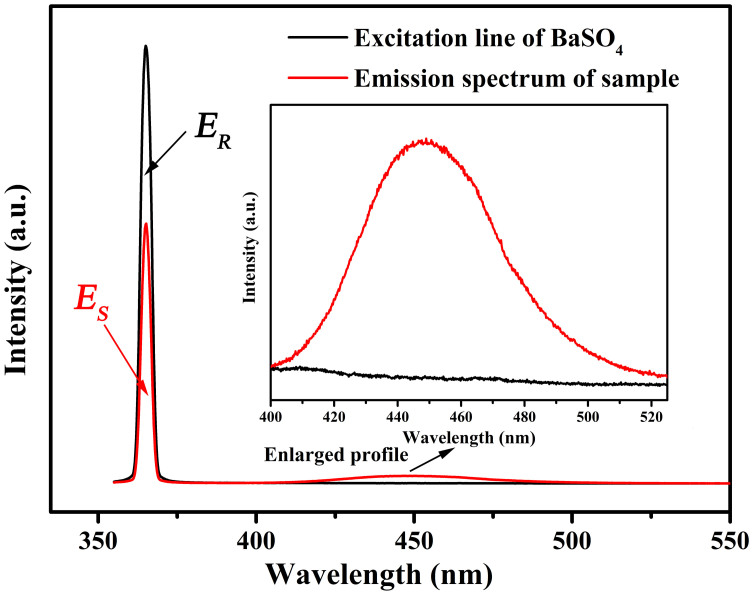
Excitation line of BaSO_4_ and emission spectrum of the KMg_4_(PO_4_)_3_:0.01Eu^2+^ phosphor collected by an integrating sphere. The inset shows the magnification of the emission spectrum.

Quantum efficiency of phosphors is another important technological parameter for practical application. The internal quantum efficiency (QE) of KMg_4_(PO_4_)_3_:0.01Eu^2+^ were measured and calculated by the following equations:^30^
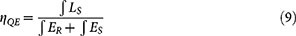
in which *L_s_* represents the luminescence emission spectrum of the sample; *E_R_* is the spectrum of the excitation light from the empty integrated sphere (without the sample); *E_S_* means the excitation spectrum for exciting the sample. As given in Figure 10, the internal QE of the KMg_4_(PO_4_)_3_:0.01Eu^2+^ phosphor is estimated to be about 50.44% under 365nm excitation. As a comparison, the internal QE of commercial BAM:Eu^2+^ phosphor is detected at the same condition and calculated to about 88.99%. The QE of KMg_4_(PO_4_)_3_:0.01Eu^2+^ can be further improved by optimization of the preparation conditions, because the QE depends closely on the prepared conditions, crystalline defects, particle size and morphology of the phosphor^31^,^32^.

**Figure 11:**
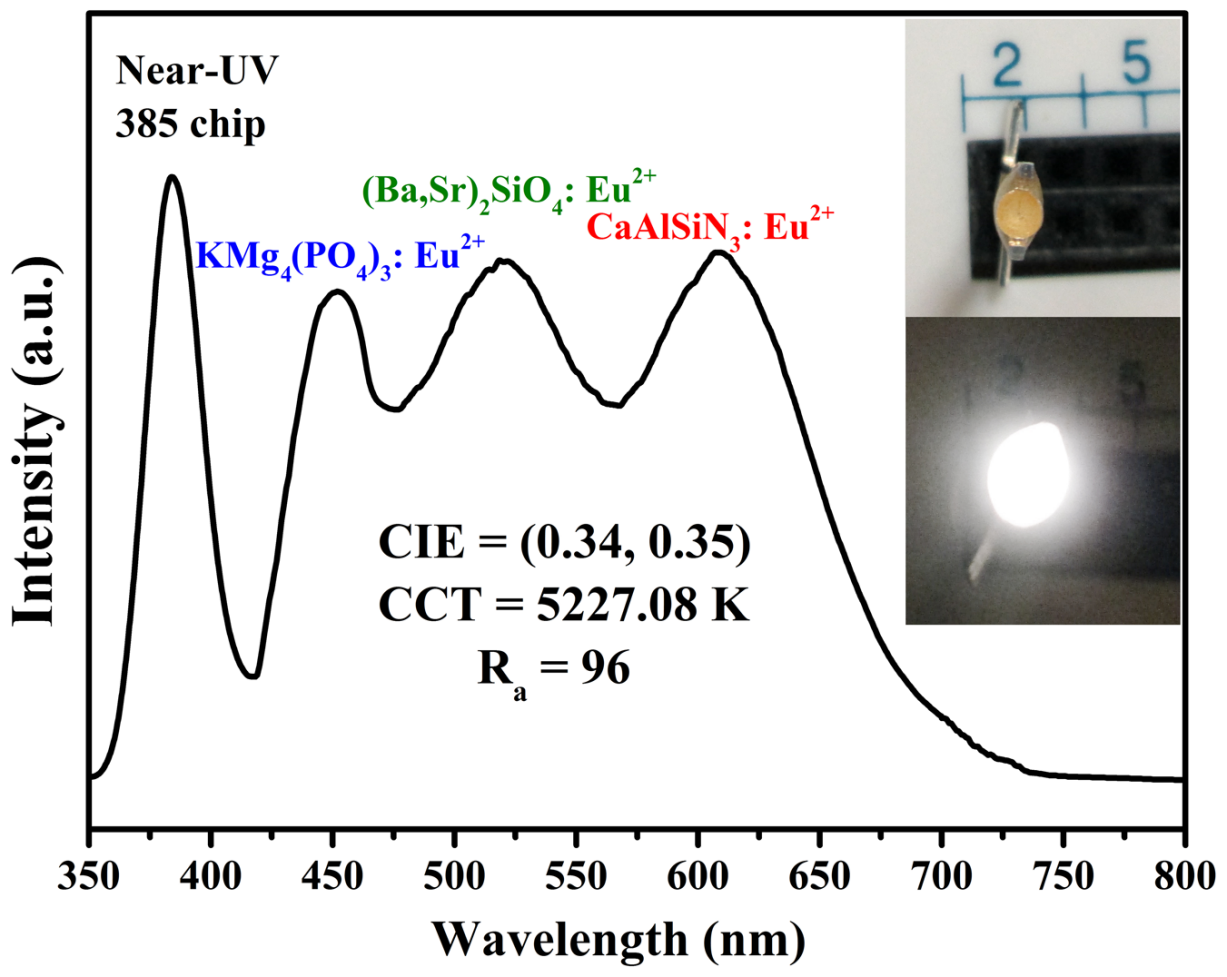
EL spectrum of a white-emitting N-UV-LED chip (385nm) comprising of KMg_4_(PO_4_)_3_:Eu^2+^ (blue), (Ba,Sr)_2_SiO_4_:Eu^2+^ (green) and CaAlSiN_3_:Eu^2+^ (red) phosphors driven by a 2mA current.

To demonstrate the potential application of KMg_4_(PO_4_)_3_:Eu^2+^ phosphor, the electroluminescent spectrum of white LED lamp which was fabricated via using N-UV LED chips (λ_max_ = 385nm) combing with blue-emitting KMg_4_(PO_4_)_3_:0.01Eu^2+^ phosphor, green-emitting (Ba,Sr)_2_SiO_4_:Eu^2+^ phosphor, and red-emitting CaAlSiN_3_:Eu^2+^ phosphor was measured as given in Figure 11 with forward bias current of 2mA. The CIE color coordinates, correlated color temperature (CCT) and color rendering index (Ra) of this fabricated WLED lamp are determined to be (0.34, 0.35), 5227.08 and 96, respectively. The Ra was decided from the full set of the first eight CRIs shown in Table 2. The appropriate CCT value (5227.08) and high Ra value (96) demonstrate that the KMg_4_(PO_4_)_3_:Eu^2+^ can be a promising candidate for a blue-emitting phosphor for application of WLEDs.

## Discussion

In conclusion, we report a systematic study on the preparation and crystal structure analysis of blue-emitting KMg_4_(PO_4_)_3_:Eu^2+^ phosphor and investigate their reflectance spectra, thermal stability, quantum efficiency and applications in N-UV LED. The phase composition and crystal structure of KMg_4_(PO_4_)_3_:Eu^2+^ were determined via the powder X-ray diffraction patterns and Rietveld refinement analysis. The optimal Eu^2+^ doping concentration in the KMg_4_(PO_4_)_3_ host is 1mol%. The critical energy transfer distance of this phosphor was calculated to be about 35.84Å and the concentration quenching mechanism is proved to be the dipole–dipole interaction. The investigation results also reveal that the as-prepared phosphor shows good thermal stability, and the internal quantum efficiency is 50.44%. The white N-UV LEDs packaged by an N-UV chip with blue-emitting KMg_4_(PO_4_)_3_:Eu^2+^, green and red-emitting phosphors generate white light with high color rendering index (Ra = 96) and an appropriate correlated color temperature (5227.08K). These results demonstrate that KMg_4_(PO_4_)_3_:Eu^2+^ is a promising blue-emitting phosphor for N-UV LEDs.

## Methods

### Materials and Synthesis

A variety of blue-emitting KMg_4_(PO_4_)_3_:*x*Eu^2+^ phosphors were prepared via a traditional high-temperature solid-state reaction. The constituent raw materials KH_2_PO_4_ (A. R.), MgO (A. R.), NH_4_H_2_PO_4_ (A. R.) and Eu_2_O_3_ (A. R.) were weighed in stoichiometric proportions and ground homogeneously in agate mortar. Firstly, the mixtures were preheated at 600°C for 2h in a muffle furnace in air to release NH_3_, CO_2_, and H_2_O. Then, the precursor was reground and heated at 1000°C for 6h in the thermal carbon reducing atmosphere (TCRA). Finally the furnace cooled to room temperature and the mixtures were ground in an agate mortar.

### Materials Characterization

X-ray powder diffraction (XRD) patterns of the final products were identified on a D8 Advance diffractometer (Bruker Corporation, Germany) with Cu Kα radiation (λ = 0.15406nm) radiation. High quality XRD data for Rietveld refinement were collected by step scanning rate (8s per step with a step size of 0.02°) over a 2θ range from 5° to 100°. The photoluminescent excitation/emission (PLE/PL) spectra were detected by a Hitachi F-4600 fluorescence spectrophotometer (Japan) equipped using a150W Xe lamp as the excitation source. The temperature-dependent luminescence properties were measured on the same spectrophotometer which was assembled with a computer-controlled electric furnace and a self-made heating attachment. The diffuse reflectance spectra were obtained by a Varian Cary-5000 UV−vis−NIR spectrophotometer attached with an integral sphere. The room-temperature luminescence decay curves were obtained from a spectrofluorometer (Horiba, Jobin Yvon TBXPS) using a tunable pulse laser radiation (nano-LED) as the excitation. Quantum efficiency was measured by a fluoromax-4 spectrofluorometer (Horiba, Jobin Yvon) with an integral sphere at room temperature.

## Figures and Tables

**Table 1:** Main parameters of processing and refinement of KMg_4_(PO_4_)_3_ host and KMg_4_(PO_4_)_3_:0.06Eu^2+^ samples

Compound	KMg_4_(PO_4_)_3_	KMg_4_(PO_4_)_3_:0.06Eu
Sp.Gr.	*Pnnm*	*Pnnm*
*a*, Å	16.3707(7)	16.3563(4)
*b*, Å	9.5627(4)	9.5570(2)
*c*, Å	6.1667(5)	6.1663(1)
*α*, °	90	90
*β*, °	90	90
*γ*, °	90	90
*V*, Å^3^	965.361(23)	963.904(67)
*2θ*-interval, °	5–90°	5–90°
*R_wp_*, %	8.86	10.54
*R_p_*, %	6.86	7.97
χ^2^	2.511	2.844

**Table 2:** Full Set of 14 CRIs and the Ra of the Fabricated WLED

R_a_	R1	R2	R3	R4	R5	R6	R7	R8	R9	R10	R11	R12	R13	R14
96	96	98	98	94	96	94	96	92	77	95	89	96	96	99
